# Supplementary material for: Machine Learning Accelerates Crystallization for Structure Determination
Source: Angew Chem Int Ed Engl. 2026 May 4;65(25):e1218503. doi: 10.1002/anie.1218503 (PMC13266958; doi:10.1002/anie.1218503)

checkCIF/PLATON report

Structure factors have been supplied for datablock(s) lcz013

THIS REPORT IS FOR GUIDANCE ONLY. IF USED AS PART OF A REVIEW PROCEDURE FOR PUBLICATION, IT SHOULD NOT REPLACE THE EXPERTISE OF AN EXPERIENCED CRYSTALLOGRAPHIC REFEREE.

No syntax errors found.   CIF dictionary   Interpreting this report

Datablock: lcz013

```
Bond precision:   C-C = 0.0106 A          Wavelength=1.54184

Cell:            a=14.1818(5)     b=13.6089(3)        c=18.2517(6)
                 alpha=90         beta=104.205(3)     gamma=90
Temperature:     100 K
```

	Calculated	Reported
Volume	3414.85(19)	3414.85(19)
Space group	P 21/c	P 1 21/c 1
Hall group	-P 2ybc	-P 2ybc
Moiety formula	C26 H17 Ag3 F18 N6 O3	C15 H3 Ag3 F18 N6, C11 H14 O3
Sum formula	C26 H17 Ag3 F18 N6 O3	C26 H17 Ag3 F18 N6 O3
Mr	1127.07	1127.06
Dx,g cm-3	2.192	2.192
Z	4	4
Mu (mm-1)	14.977	14.977
F000	2168.0	2168.0
F000'	2178.48	
h,k,lmax	16,16,21	16,16,21
Nref	6023	6008
Tmin,Tmax	0.125,0.224	0.373,1.000
Tmin'	0.020	

```
Correction method= # Reported T Limits: Tmin=0.373 Tmax=1.000
AbsCorr = MULTI-SCAN
```

Data completeness= 0.998 Theta(max)= 66.555

R(reflections)= 0.0673(5173) wR2(reflections)= 0.1804(6008)

S = 1.016 Npar= 508

The following ALERTS were generated. Each ALERT has the format
test-name_ALERT_alert-type_alert-level.
Click on the hyperlinks for more details of the test.

Alert level A

PLAT971_ALERT_2_A Check Calcd Resid. Dens. 1.40Ang From O00E 4.94 eA-3

Author Response: This Alert is due to presence of residual density in the presence of heavy metal atom (Ag).

PLAT971_ALERT_2_A Check Calcd Resid. Dens. 1.18Ang From C014 4.37 eA-3

Author Response: This Alert is due to presence of residual density in the presence of heavy metal atom (Ag).

PLAT971_ALERT_2_A Check Calcd Resid. Dens. 1.78Ang From C014 3.75 eA-3

Author Response: This Alert is due to presence of residual density in the presence of heavy metal atom (Ag).

Alert level B

PLAT973_ALERT_2_B Check Calcd Positive Resid. Density on Ag02 1.94 eA-3

Author Response: This Alert is due to presence of residual density in the presence of heavy metal atom (Ag).

Alert level C

```
DIFMX02_ALERT_1_C  The maximum difference density is > 0.1*ZMAX*0.75
            The relevant atom site should be identified.
PLAT042_ALERT_1_C Calc. and Reported MoietyFormula Strings  Differ     Please Check
              Calc: C26 H17 Ag3 F18 N6 O3
              Rep.: C15 H3 Ag3 F18 N6, C11 H14 O3
PLAT094_ALERT_2_C Ratio of Maximum / Minimum Residual Density ....       2.68 Report
PLAT097_ALERT_2_C Large Reported Max.  (Positive) Residual Density       4.68 eA-3
PLAT213_ALERT_2_C Atom F00T            has ADP max/min Ratio .....        3.7 prolat
PLAT342_ALERT_3_C Low Bond Precision on  C-C Bonds ...............    0.01062 Ang.
PLAT906_ALERT_3_C Large K Value in the Analysis of Variance ......      3.457 Check
```

```
PLAT911_ALERT_3_C Missing FCF Refl Between Thmin & STh/L=    0.595         15 Report
                5  8  0,   0  0  2, -14  8  2, -13  8  2,   3  7  5,   5 13  9,
                5 12 10, -15  4 12,   2  4 12,   4  8 13,   3  8 14,   3  7 15,
                3  8 15,   3  7 17,   2  7 18,
PLAT971_ALERT_2_C Check Calcd Resid. Dens.  0.24Ang From Ag03           2.29 eA-3
```

Author Response: This Alert is due to presence of residual density in the presence of heavy metal atom (Ag).

```
PLAT971_ALERT_2_C Check Calcd Resid. Dens.  0.25Ang From Ag01           2.08 eA-3
```

Author Response: This Alert is due to presence of residual density in the presence of heavy metal atom (Ag).

```
PLAT971_ALERT_2_C Check Calcd Resid. Dens.  0.79Ang From N00O           2.02 eA-3
```

Author Response: This Alert is due to presence of residual density in the presence of heavy metal atom (Ag).

```
PLAT971_ALERT_2_C Check Calcd Resid. Dens.  1.57Ang From Ag01           1.74 eA-3
```

Author Response: This Alert is due to presence of residual density in the presence of heavy metal atom (Ag).

```
PLAT971_ALERT_2_C Check Calcd Resid. Dens.  0.52Ang From N009           1.67 eA-3
```

Author Response: This Alert is due to presence of residual density in the presence of heavy metal atom (Ag).

```
PLAT971_ALERT_2_C Check Calcd Resid. Dens.  1.12Ang From Ag01           1.67 eA-3
```

Author Response: This Alert is due to presence of residual density in the presence of heavy metal atom (Ag).

```
PLAT971_ALERT_2_C Check Calcd Resid. Dens.  1.18Ang From Ag03           1.60 eA-3
```

Author Response: This Alert is due to presence of residual density in the presence of heavy metal atom (Ag).

```
PLAT972_ALERT_2_C Check Calcd Resid. Dens.  0.86Ang From Ag02          -1.87 eA-3
PLAT972_ALERT_2_C Check Calcd Resid. Dens.  0.87Ang From Ag02          -1.76 eA-3
PLAT972_ALERT_2_C Check Calcd Resid. Dens.  0.79Ang From Ag03          -1.68 eA-3
PLAT972_ALERT_2_C Check Calcd Resid. Dens.  0.86Ang From Ag03          -1.61 eA-3
PLAT972_ALERT_2_C Check Calcd Resid. Dens.  0.80Ang From Ag02          -1.58 eA-3
PLAT972_ALERT_2_C Check Calcd Resid. Dens.  0.83Ang From Ag01          -1.53 eA-3
PLAT972_ALERT_2_C Check Calcd Resid. Dens.  0.79Ang From Ag01          -1.52 eA-3
```

Alert level G

```
PLAT003_ALERT_2_G Number of Uiso or U(i,j)  Restrained non-H-Atoms          2 Report
PLAT072_ALERT_2_G SHELXL First  Parameter in WGHT  Unusually Large       0.15 Report
PLAT186_ALERT_4_G The CIF-Embedded .res File Contains ISOR Records          1 Report
PLAT242_ALERT_2_G Low    'MainMol' Ueq as Compared to Neighbors of       C00X Check
PLAT242_ALERT_2_G Low    'MainMol' Ueq as Compared to Neighbors of       C00Y Check
PLAT242_ALERT_2_G Low    'MainMol' Ueq as Compared to Neighbors of       C017 Check
PLAT242_ALERT_2_G Low    'MainMol' Ueq as Compared to Neighbors of       C01A Check
PLAT242_ALERT_2_G Low    'MainMol' Ueq as Compared to Neighbors of       C01G Check
PLAT242_ALERT_2_G Low    'MainMol' Ueq as Compared to Neighbors of       C01H Check
PLAT434_ALERT_2_G Short Inter HL..HL Contact F00C     ..F00T     .       2.78 Ang.
                                            -x,-1/2+y,3/2-z  =      2_546 Check
PLAT434_ALERT_2_G Short Inter HL..HL Contact F00H     ..F00P     .       2.79 Ang.
                                              x,1/2-y,1/2+z  =      4_566 Check
PLAT720_ALERT_4_G Number of Unusual/Non-Standard Labels ..........         73 Note
              Ag01    Ag02    Ag03    F004    F005    F006    F007    F008
              N009    F00A    F00C    F00D    O00E    F00F    F00G    F00H
              F00I    O00J    F00K    N00L    N00M    N00N    N00O    F00P
              N00Q    F00R    F00S    F00T    F00U    C00V    H00V    C00W
              C00X    C00Y    C00Z    C010    H010    C011    C012    H012
              C013    C014    H01A    H01B    C015    H015    C016    H016
              C017    C018    C019    C01A    C01B    C01C    C01D    H01C
              H01D    H01E    C01E    H01F    C01F    C01G    C01H    C01I
              C01J    H01G    H01H    H01I    C01K    H01J    H01K    H01L
              O01M
PLAT860_ALERT_3_G Number of Least-Squares Restraints .............         12 Note
PLAT883_ALERT_1_G Absent Datum for _atom_sites_solution_primary ..     Please Do !
PLAT909_ALERT_3_G Percentage of I>2sig(I) Data at Theta(Max) Still        76% Note
PLAT933_ALERT_2_G Number of HKL-OMIT Records in Embedded .res File          4 Note
               -9  0 22,  -9  1 22,  -9  2 22, -11  3 19,
PLAT941_ALERT_3_G Average HKL Measurement Multiplicity ...........        2.7 Low
PLAT969_ALERT_5_G The 'Henn et al.' R-Factor-gap value ...........      3.410 Note
              Predicted wR2: Based on SigI**2  5.29 or SHELX Weight 17.76
PLAT978_ALERT_2_G Number C-C Bonds with Positive Residual Density.          0 Info
```

---

3 **ALERT level A** = Most likely a serious problem - resolve or explain
1 **ALERT level B** = A potentially serious problem, consider carefully
22 **ALERT level C** = Check. Ensure it is not caused by an omission or oversight
19 **ALERT level G** = General information/check it is not something unexpected

3 ALERT type 1 CIF construction/syntax error, inconsistent or missing data
33 ALERT type 2 Indicator that the structure model may be wrong or deficient
6 ALERT type 3 Indicator that the structure quality may be low
2 ALERT type 4 Improvement, methodology, query or suggestion
1 ALERT type 5 Informative message, check

It is advisable to attempt to resolve as many as possible of the alerts in all categories. Often the minor alerts point to easily fixed oversights, errors and omissions in your CIF or refinement strategy, so attention to these fine details can be worthwhile. It is up to the individual to critically assess their own results and, if necessary, seek expert advice.

Validation response form

Please find below a validation response form (VRF) that can be filled in and pasted into your CIF.

```
# start Validation Reply Form
_vrf_DIFMX02_lcz013
;
PROBLEM: The maximum difference density is > 0.1*ZMAX*0.75
RESPONSE: ...
;
_vrf_PLAT042_lcz013
;
PROBLEM: Calc. and Reported MoietyFormula Strings  Differ     Please Check
RESPONSE: ...
;
_vrf_PLAT094_lcz013
;
PROBLEM: Ratio of Maximum / Minimum Residual Density ....       2.68 Report
RESPONSE: ...
;
_vrf_PLAT097_lcz013
;
PROBLEM: Large Reported Max.  (Positive) Residual Density       4.68 eA-3
RESPONSE: ...
;
_vrf_PLAT213_lcz013
;
PROBLEM: Atom F00T            has ADP max/min Ratio .....        3.7 prolat
RESPONSE: ...
;
_vrf_PLAT342_lcz013
;
PROBLEM: Low Bond Precision on  C-C Bonds ..............    0.01062 Ang.
RESPONSE: ...
;
_vrf_PLAT906_lcz013
;
PROBLEM: Large K Value in the Analysis of Variance ......      3.457 Check
RESPONSE: ...
;
_vrf_PLAT911_lcz013
;
PROBLEM: Missing FCF Refl Between Thmin & STh/L=    0.595         15 Report
RESPONSE: ...
;
_vrf_PLAT972_lcz013
;
PROBLEM: Check Calcd Resid. Dens.  0.86Ang From Ag02           -1.87 eA-3
RESPONSE: ...
;
# end Validation Reply Form
```

PLATON version of 17/09/2025; check.def file version of 17/09/2025

---

duplicate check

No duplication found

Datablock lcz013 - ellipsoid plot

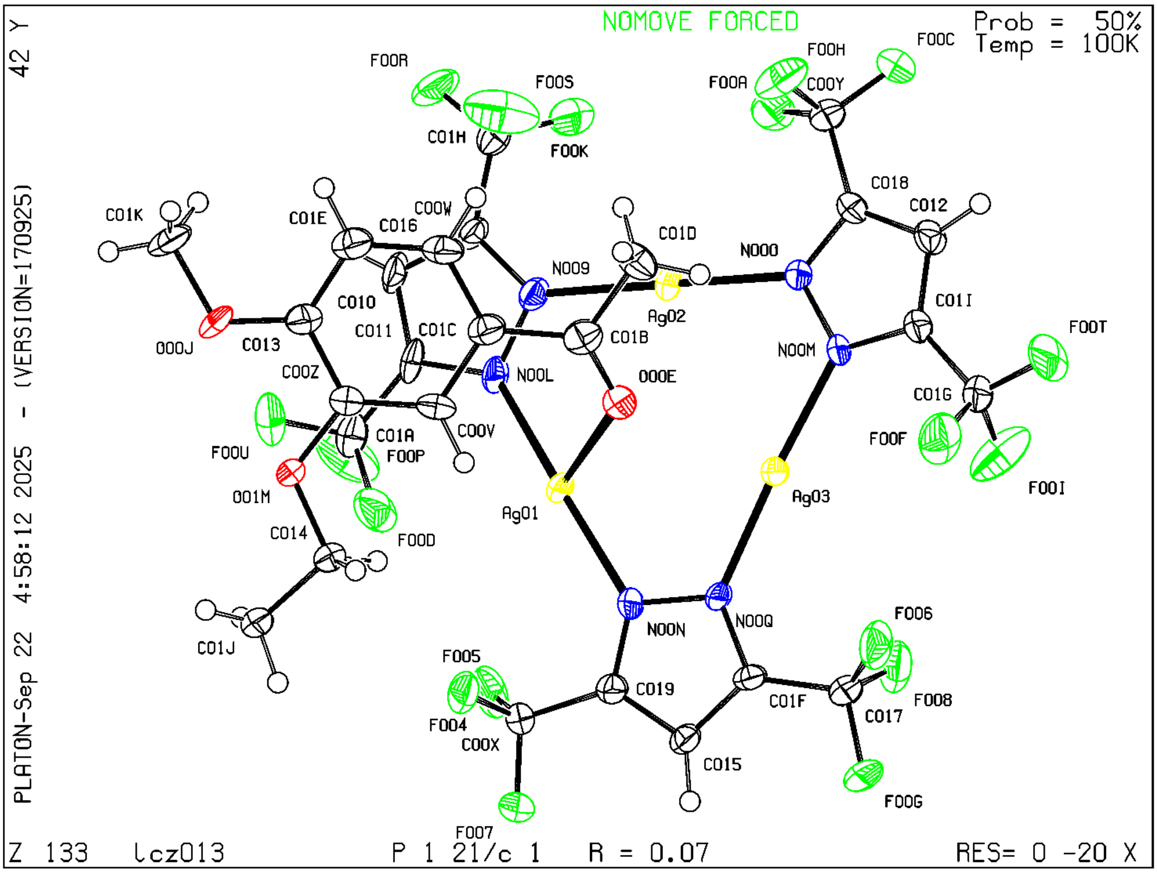

Supplement: Supplementary file 2 — Supporting File 2: anie72462‐sup‐0002‐DataFile.zip. [file ANIE-65-e1218503-s002.zip › supplementary_materials_crystallographic_data submit.zip!/cif_and_checkcif(1-114)/Ag3Pz3_13/Ag3Pz3_13_checkcif.pdf]
